# Leaky gut and inflammatory biomarkers in a medication overuse headache model in male rats

**DOI:** 10.55730/1300-0144.5763

**Published:** 2023-10-25

**Authors:** Doğa VURALLI, Hale GÖK DAĞIDIR, Elif ABBASOĞLU TOPA, Hayrunnisa BOLAY BELEN

**Affiliations:** 1 Department of Neurology and Algology, Faculty of Medicine, Gazi University, Ankara, Turkiye; 2 Neuroscience and Neurotechnology Center of Excellence (NÖROM), Gazi University, Ankara, Turkiye; 3 Neuropsychiatry Center, Gazi University, Ankara, Turkiye

**Keywords:** Medication overuse headache, leaky gut, inflammation, occludin, lipopolysaccharide binding protein, high mobility group box protein 1

## Abstract

**Background/aim:**

Medication overuse is common among chronic migraine patients and nonsteroidal antiinflammatory drugs (NSAIDs) are the most frequently overused drugs. The pathophysiological mechanisms underlying medication overuse headache (MOH) are not completely understood. Intestinal hyperpermeability and leaky gut are reported in patients using NSAIDs. The aim of the study is to investigate the role of leaky gut and inflammation in an MOH model MOH model in male rats.

**Methods:**

The study was conducted in male Sprague Dawley rats. There were two experimental groups. The first group was the chronic NSAID group in which the rats received mefenamic acid (n = 8) for four weeks intraperitoneally (ip) and the second group was the vehicle group (n = 8) that received 5% dimethyl sulfoxide+sesame oil (ip) for 4 weeks. We assessed spontaneous pain-like behavior, periorbital mechanical withdrawal thresholds, and anxiety-like behavior using an elevated plus maze test. After behavioral testing, serum levels of occludin and lipopolysaccharide-binding protein (LBP) and brain levels of IL-17, IL-6, and high mobility group box 1 protein (HMGB1) were evaluated with ELISA.

Results: Serum LBP and occludin levels and brain IL-17 and HMGB1 levels were significantly elevated in the chronic NSAID group compared to its vehicle (p = 0.006, p = 0.016, p = 0.016 and p = 0.016 respectively) while brain IL-6 levels were comparable (p = 0.67) between the groups. The chronic NSAID group showed pain-like and anxiety-like behavior in behavioral tests. Brain IL-17 level was positively correlated with number of head shakes (r = 0.64, p = 0.045), brain IL-6 level was negatively correlated with periorbital mechanical withdrawal thresholds (r = −0.71, p = 0.049), and serum occludin level was positively correlated with grooming duration (r = 0.73, p = 0.032) in chronic NSAID group.

**Conclusion:**

Elevated serum occludin and LBP levels and brain IL-17 and HMGB1 levels indicate a possible role of leaky gut and inflammation in an MOH model in male rats. Additionally, a significant correlation between pain behavior and markers of inflammation and intestinal hyperpermeability, supports the role of inflammation and leaky gut in MOH pathophysiology.

## 1. Introduction

Overuse of analgesics is common in chronic headache disorders, especially in chronic migraine. The frequent use of analgesics leads to migraine chronification and approximately half of the chronic migraine patients who are admitted to headache centers also suffer from medication overuse headache (MOH) [1]. MOH is seen in 1%–2% of the general population [2]. Even though MOH is a common entity in chronic migraine, the mechanisms underlying MOH needs to be elucidated further.

Animal model studies increased our knowledge about the pathophysiology of MOH. Even though NSAIDs are the most commonly used analgesics in MOH, clinically relevant animal models using NSAIDs are scarce [3]. Enhanced cortical excitability [3–6] and central sensitization [3] have been shown in MOH models. Neuroinflammation has been shown in many pain disorders including migraine [7]. Several cytokines have been associated with migraine [8–10], suggesting an existing inflammation in migraine patients. Recently, a potential role of inflammation has been suggested in an MOH model in female rats [11].

The gut-brain axis consists of a bidirectional interaction and gained interest in recent years in neurological research. The main component of the intestinal barrier is the tight junctions connecting the epithelial cells. Occludin was the first identified integral membrane protein of epithelial tight junctions. Occludin has an essential role in maintaining intestinal barrier integrity. Additionally, factors such as mucins, immunoglobulins, and cytokines support the intestinal barrier. A disturbance in the intestinal epithelium or the factors supporting the intestinal barrier may increase intestinal permeability and may cause a “leaky gut” [12] and in this condition, tight junction proteins can be detected in the blood [13, 14]. The intestinal barrier prevents the passage of toxic materials and microorganisms from the lumen to the bloodstream. In the case of a leaky gut, intestinal barrier integrity and function are impaired, resulting in the passage of pathogens, toxic and inflammatory substances into the blood [12]. Permeability changes and leaky gut are reported in patients receiving NSAID treatment [15–18] and chronic intake of NSAIDs have been shown to alter intestinal barrier function and result in intestinal inflammation [15]. Leaky gut and increased intestinal permeability are associated with irritable bowel syndrome (IBS) [19] and IBS symptoms are shown to be significantly more frequent in patients with chronic migraine and MOH [20].

Translocation of bacteria and bacterial products to systemic circulation occurs when there is a dysfunction of the intestinal barrier. Lipopolysaccharide (LPS) is a pathogen-associated molecular pattern found in the outer membrane of gram-negative bacteria and can be shed from the bacterial membrane [21]. Disrupted intestinal barrier results in LPS transport into the systemic circulation. LPS is bound mostly to LPS binding protein (LBP) in blood and activates the TLR4 receptor on immune cells and starts an inflammatory response with the release of proinflammatory cytokines including IL-6 [22]. TLR4 binding to LPS needs CD14 as a cofactor. LBP transports LPS to CD14. This cascade ends up activating NF-κB transcription pathway [23] that regulates the release of various proinflammatory cytokines. LBP is an acute-phase protein synthesized in the liver and normally its serum level is very low but increases as a result of inflammation [21].

In a nitroglycerin (NTG) migraine animal model, it was shown that IL-17 crosses the blood brain barrier and provokes neuroinflammation, supporting the role of neuroinflammation in migraine [24]. Another inflammatory mediator implied in migraine is IL-6, a proinflammatory cytokine that is shown to be increased in the internal jugular blood of migraine patients during a migraine attack [10]. Additionally, IL-6 levels were demonstrated to be correlated with migraine headache frequency [25]. Application of IL-6 to the dura of rats and mice is shown to cause facial [26–28] and hindpaw [26, 29] allodynia. When IL-6 is applied to the dura, the rats even responded to subthreshold triggers such as calcitonin gene-related peptide (CGRP) and decreased pH, showing an increased susceptibility to triggers [26, 27]. TLR4 is also activated by high mobility group box protein 1 (HMGB1), a damage-associated molecular pattern (DAMP). HMGB1 is an intranuclear protein and when it is released and transported to the extracellular space, it plays a role as a proinflammatory mediator. A possible role of leaky gut and associated inflammation including HMGB1 was suggested in a MOH model using piroxicam in female rats [11]. The role of cytokines above and HMGB1 in a MOH model in male rats remains to be explored.

We aimed to investigate whether serum levels of occludin and LBP and brain levels of IL-17, IL-6, and HMGB1 are elevated indicating a leaky gut and inflammation in a MOH model induced by intraperitoneal mefenamic acid administration in male rats. We also aimed to evaluate pain and anxiety-like behavior and periorbital mechanical sensitivity in this MOH model and assess possible correlations between spontaneous and evoked animal behaviors and inflammatory markers.

## 2. Materials and methods

Experiments were performed using 9–10-week-old, 200–250 gr, 16 male Sprague Dawley rats at Gazi University. Male rats were used in the experiments to avoid the influence of different estrous cycle phases on pain thresholds and to evaluate the role of leaky gut and associated inflammation in an MOH model in male rats for the first time. Rats were housed in transparent plexiglass cages in a climate-controlled environment with ad libitum access to food and water. The cages were exposed to 12 h of daylight and 12 h of darkness. All procedures were conducted in accordance with Turkish Animal Welfare Act and the institutional review board approved the study.

Mefenamic acid (Sigma-Aldrich, USA), an NSAID, or its vehicle, 5% dimethyl sulfoxide (DMSO) (Sigma-Aldrich, USA) + sesame oil (Sigma-Aldrich, USA) was used. The animals received either mefenamic acid 20 mg/kg (n = 8) or its vehicle 1 mL/kg (n = 8) intraperitoneally for 4 weeks as previously reported in an MOH model [3]. Animals were assigned to treatment groups randomly and body weight was monitored weekly. Behavioral testing was performed at the same time frame each day. The spontaneous behavior of the rats was recorded for 10 min in transparent cages. Grooming, freezing, immobility, and head shakes during the 10 minutes were evaluated. Additionally, “up and down method” beginning with a force of 4 g was used to assess periorbital mechanical withdrawal thresholds [30]. Rats were placed on the 40 cm high platforms and von Frey filaments were used to evaluate the mechanical withdrawal thresholds once they adapted to the platforms. A positive response was noted if ipsilateral head grooming and avoidance of rats from the filament were observed. Anxiety-like responses were evaluated with elevated plus maze [3, 31]. After placing the rats at the center of the plus-shaped maze facing the same closed arm, the behavior of the rats was recorded with a video camera system for 5 min. The durations spent in open and closed arms and the number of open and closed arm entries were assessed.

**Figure 1:**
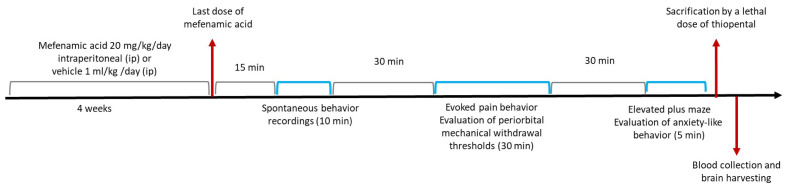
The experimental timeline design of the study.

After the completion of the behavioral testing, 2 h after the last dose of mefenamic acid, the animals were sacrificed by a lethal dose of thiopental (50 mg/kg) and 7–8 cc blood were collected with cardiac puncture and following cervical dislocation and decapitation, the brains were harvested. The blood samples were centrifuged to obtain the serum (20 min 1000*g at 2–8 °C) according to enzyme-linked immuno sorbent assay (ELISA) kit protocol. The serum samples and the brains were stored at −80 °C. The experimental timeline design of the study is given in Figure 1.

### 2.1. Enzyme-linked immuno sorbent assay (ELISA)

High-sensitivity ELISA rat kits were used to measure brain IL-17, IL-6, and HMGB1, serum LBP and occludin levels. Brain IL-17 (MBS2022678) and IL-6 levels (MBS2021530) were measured with a sandwich-type rat ELISA kit from MyBiosource Inc., USA. Serum LBP (E-EL-R0589) and occludin levels (E-EL-R2503) and brain tissue HMGB1 (E-EL-R0505) were measured with sandwich-type rat ELISA kits from Elabscience Biotechnology Inc., Houston, TX, USA. The coefficient of variation for repeatability was <10%.

The brain tissues were homogenized on ice before centrifugation in accordance with the instructions of the manufacturer. The solutions for the kits were prepared with analytical deionized water and all reagents were brought to room temperature as specified in the ELISA kit procedures. Standards and samples were put to the wells in the microliters defined in each kit. The solutions were added, the number of washes and the incubation times were advanced according to the kit procedures. During the incubation period, the plate wells were covered with a sealing film. Following the last wash, tetramethylbenzidine was added to each plate well for a blue color reaction. A microplate reader was used to measure the optical densities of the wells at 450 nm after adding an acidic stop solution to the wells with a rapid change of color from blue to yellow. Serum and tissue homogenate concentrations were calculated in accordance with the standard graph. Combiwash Human ELISA plate washer (Combiwash Germany Chromate Awareness Technology, USA) and Chromate reader (Combiwash Human Diagnostics Worldwide, Germany) were used during the measurements.

### 2.2. Statistics and study design

The experiments and analyses were performed in a blinded fashion. The researchers who performed the behavioral tests and ELISA were blinded to the experimental groups/conditions. None of the animals died during the treatment period. Analyses were performed using IBM SPSS 22.0 for Windows software (USA). Data were shown as mean ± standard deviation and bar graphs. The Kolmogorov-Smirnov test was used to assess normalisation of data. The analysis of continuous variables was performed using an independent samples t-test. Pearson’s correlation analysis was used to assess possible correlations between the spontaneous or evoked behavior of animals and brain IL-17, IL-6, and HMGB1 levels and serum LBP and occludin levels. A p-value of 0.05 or less was considered statistically significant.

## 3. Results

**Figure 2:**
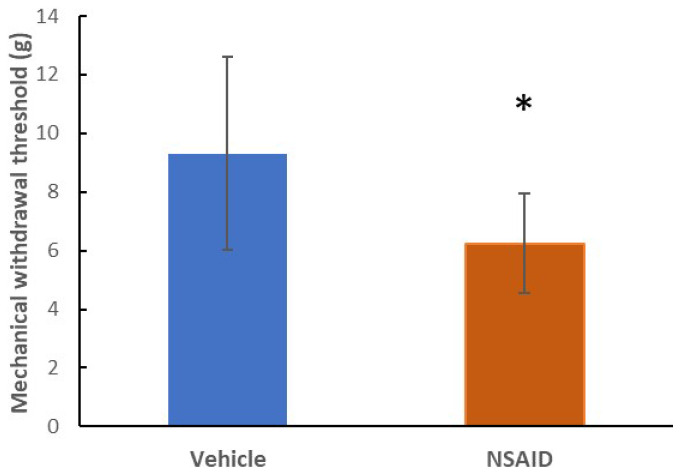
The mechanical withdrawal thresholds were significantly lower (p = 0.035) in the NSAID group compared to the vehicle group. *p < 0.05 vs. controls.

**Figure 3:**
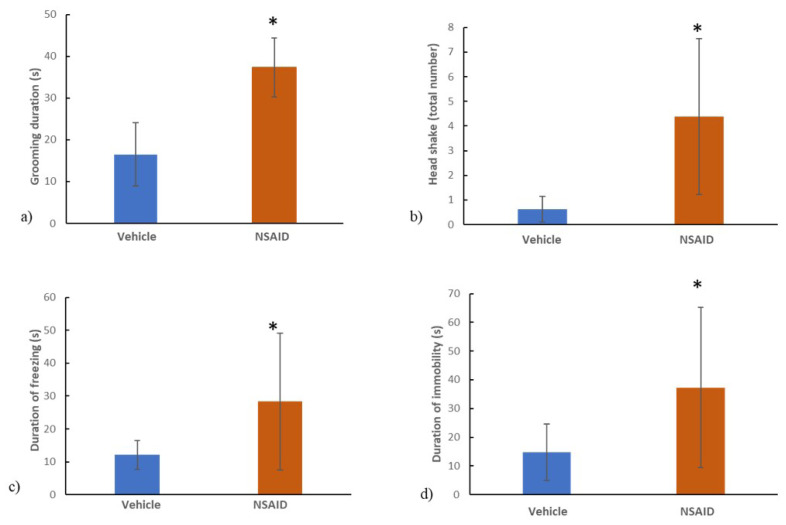
a) The grooming duration b) the number of headshakes c) the duration of freezing d) the duration of immobility were significantly higher in the NSAID group compared to the vehicle (p = 0.0001, p = 0.005, p = 0.048, p = 0.049 respectively). *p < 0.05 vs. controls.

The chronic NSAID group (9.1 ± 3.3 g) had significantly lower mechanical withdrawal thresholds (p = 0.035) compared to its vehicle (6.3 ± 1.7 g) (Figure 2). The grooming duration (16.5 ± 7.6 s in the vehicle group and 37.3 ± 7.9 s in the chronic NSAID group) (Figure 3a), the number of head shakes (0.6 ± 0.5 in the vehicle group and 4.4 ± 3.2 in the chronic NSAID group) (Figure 3b), durations of freezing (12.1 ± 4.4 s in the vehicle group and 28.4 ± 20.8 s in the chronic NSAID group) (Figure 3c) and immobility (14.8 ± 9.8 s in the vehicle group and 37.3 ± 27.9 s in the chronic NSAID group) (Figure 3d) were significantly higher in the chronic NSAID group compared to the vehicle (p = 0.0001, p = 0.005, p = 0.048 and p = 0.049 respectively).

**Figure 4:**
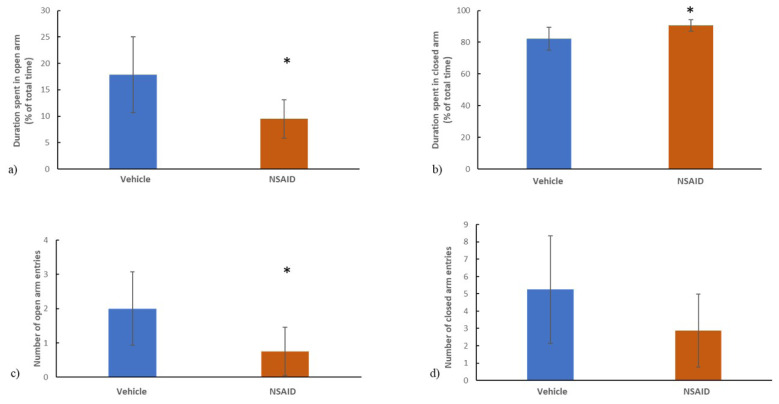
In the elevated plus maze test, a) duration spent in open arms was lower and b) duration spent in closed arms was higher c) the number of open arm entries was lower in the NSAID group compared to the vehicle (p = 0.011, p = 0.011 and p = 0.015, respectively) and d) the number of closed arm entries were comparable between the two groups (p = 0.095). *p < 0.05 vs. controls.

In an elevated plus maze test, duration spent in open arms was 17.9% ± 7.2% in the vehicle group and 9.5% ± 3.6% in the chronic NSAID group whereas duration spent in closed arms was 82.1 ± 7.2% in the vehicle group and 90.5% ± 3.6% in the chronic NSAID group. Duration spent in open arms was significantly decreased (p = 0.011) (Figure 4a) and duration spent in closed arms was significantly increased (p =0.011) in the chronic NSAID group compared to vehicle (Figure 4b). The number of open-arm entries was significantly lower (p = 0.015) in the chronic NSAID group (0.75 ± 1.71) compared to its vehicle (2.0 ± 1.1) (Figure 4c) while the number of closed-arm entries was comparable (p = 0.095) between two groups (5.25 ± 3.1 in the vehicle group and 2.9 ± 2.1 in the chronic NSAID group) (Figure 4d).

**Figure 5:**
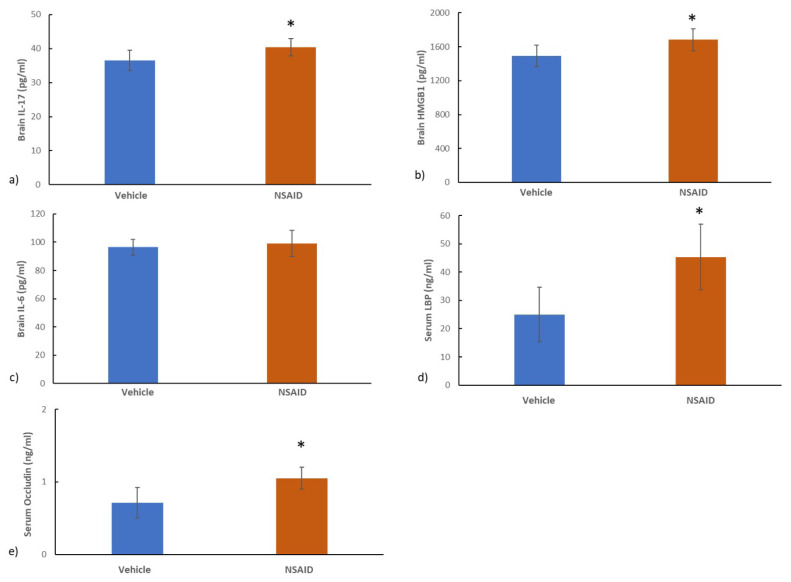
a) Brain IL-17 and b) HMGB1 levels were significantly higher in the NSAID group compared to its vehicle (p = 0.016 and p = 0.016, respectively). c) Brain IL-6 levels were similar between the two groups (p = 0.67). d) Significantly greater serum LBP and e) occludin levels were found in the NSAID group compared to the vehicle (p = 0.006 and p = 0.016, respectively). *p < 0.05 vs. controls.

Brain IL-17 (36.5 ± 3.0 pg/mL in the vehicle group and 40.4 ± 2.3 pg/mL in the chronic NSAID group) (Figure 5a) and HMGB1 levels (1493.5 ± 128.0 pg/mL in the vehicle group and 1683.6 ± 128.3 pg/mL in the chronic NSAID group) (Figure 5b) were significantly higher in the chronic NSAID acid group compared to its vehicle (p = 0.016 and p = 0.016, respectively) while brain IL-6 levels (96. 4 ± 5.7 pg/mL in the vehicle group and 99.1 ± 9.3 pg/mL in the chronic NSAID group) (Figure 5c) were comparable between the two groups (p = 0.67) (Figure 5). Mean serum LBP was 25.0 ± 9.6 ng/mL in the vehicle group and 45.3 ± 11.5 ng/mL in the chronic NSAID group (Figure 5d). The mean serum occludin level was 0.7 ± 0.2 ng/mL in the vehicle group and 1.1 ± 0.2 ng/mL in the chronic NSAID group (Figure 5e). Serum LBP and occludin levels were significantly higher in the chronic NSAID group compared to its vehicle (p = 0.006 and p = 0.016, respectively).

Brain IL-17 was positively correlated with a number of head shakes (r = 0.64, p = 0.045) in chronic NSAID group. Brain IL-6 was negatively correlated with periorbital mechanical withdrawal thresholds (r = −0.71, p = 0.049), as periorbital mechanical withdrawal thresholds decreased, brain IL-6 levels increased in chronic NSAID group. Serum occludin level was positively correlated with grooming duration in chronic NSAID group (r = 0.73, p = 0.032)

## 4. Discusssion

We showed that brain IL-17 and HMGB1 levels and serum occludin and LBP levels are increased in male rats that received chronic NSAID. Chronic NSAID resulted in higher brain IL-17 and HMGB1 levels and serum occludin and LBP levels compared to its vehicle. In this MOH rat model, chronic NSAID exposure resulted in mechanical hypersensitivity and pain-like behavior that were compatible with medication-overuse headaches. Anxiety-like behavior was also observed in this MOH model consistent with the common psychiatric comorbidities of chronic headaches.

We showed in an MOH model that brain IL-17 level was positively correlated with a number of head shakes and brain IL-6 level was negatively correlated with periorbital mechanical withdrawal thresholds, as periorbital mechanical withdrawal thresholds decreased, brain IL-6 levels increased. Grooming duration was positively correlated with serum occludin levels. A significant correlation between the pain-related clinical features and inflammatory markers and factors indicative of increased intestinal permeability, strongly suggests a possible role of inflammation and leaky gut in the pathophysiology of MOH.

IL-17 triggers the release of various cytokines, chemokines, and matrix metalloproteinases [32]. It has an important role in chronic inflammation and autoimmune diseases [33]. In a chronic NTG migraine model, IL-17 was shown to cross the blood-brain barrier and induce neuroinflammation [24]. Additionally, elevated plasma IL-17 level has been shown in a sound stress migraine model [34]. We showed elevated brain IL-17 levels in male rats receiving chronic NSAID, suggesting a role of inflammation in MOH.

Head shakes are among the spontaneous behaviors exacerbated in response to nociception. The number of head shakes is increased in an MOH model [3] and a nitroglycerin-induced migraine model [35] and is associated with increased cortical spreading depression waves [36]. The correlation between the head shake behavior of rats with IL-17 shows that this pain-like spontaneous behavior is associated with inflammation supporting the role of inflammation in MOH. IL-17 is also shown to be associated with cognitive deficits [37] which could be one of the causes underlying cognitive symptoms in chronic headaches such as MOH. LPS has been shown to trigger proinflammatory cascades via plasma membrane proteins such as TLR4 leading to the production of proinflammatory cytokines. IL-17A was shown to be increased in LPS-induced systemic inflammation [38].

IL-6 is produced by B cells [39] whereas IL-17 is mostly produced by T helper cells (Th17 cells) [40]. No significant difference in brain IL-6 levels between chronic NSAID and the vehicle groups whereas a significant difference between brain IL-17 levels between the two groups may suggest a stronger and more critical role of T cell-mediated inflammation in this MOH model.

HMGB1 is a proinflammatory mediator in the extracellular space and also a strong nociceptive molecule. In a MOH model in female rats using oral piroxicam, higher brain HMGB1 levels were shown to be positively correlated with headache behavior and elevated serum LBP levels [11]. Increased serum HMGB1 levels is associated with headache severity and paracetamol unresponsiveness in COVID-19 patients with headache [41]. We have also shown elevated brain HMGB1 levels supporting the role of innate immune system involvement and inflammation in this MOH model in male rats.

Occludin as a component of tight junctions plays an essential role in the maintenance of tight junctions and epithelial barriers and thus intestinal integrity. Disruption of this structure results in permeability changes and detection of soluble occludin levels in the circulation in the presence of a leaky gut. While the intestinal barrier regulates the absorption of nutrients and water from the lumen, it prevents the transmission of toxic materials and microorganisms to the circulation. The toxins entering the bloodstream may lead to a low chronic inflammatory response. NSAID intake by patients and healthy subjects has been reported to promote altered intestinal barrier function and hypermotility [13] and chronic intake of NSAID has been shown to cause leaky gut [15–18]. LBP is used to assess intestinal permeability and it is a promising biomarker since it has been shown to be unaffected by age, sex and body-mass index [42], with a long half-life and a low intraindividual variability [43].

We have shown elevated serum occludin and LBP levels similar to a previous female rat MOH model [11] and a correlation between serum occludin levels and pain-related grooming behavior in this chronic NSAID-induced MOH model in male rats. Chronic use of an NSAID resulted in trigeminal nociception that was correlated with inflammatory biomarkers, IL-17 and HMGB1 and factors suggesting intestinal hyperpermeability, occludin and LBP supporting the role of inflammation, and leaky gut in MOH pathophysiology. Sex-related differences can be observed due to hormonal factors in animal studies however, we observed pain-like behavior and increased serum markers associated with leaky gut and brain markers for inflammation in male rats similar to the study in female rats [11]. Future studies in patients with MOH are required to confirm whether intestinal hyperpermeability and LPS leak into the bloodstream is an accompanying feature of MOH and to assess the biomarker potential of occludin and LBP.

The strengths of the study are the demonstration of leaky gut and neuroinflammation due to long-term use of an NSAID in male rats. In addition, we have also shown that mefenamic acid, an NSAID, other than piroxicam [11], can also cause LPS leak into the systemic circulation and inflammatory response in the brain. One limitation of the study is that chronic migraine and accompanying MOH are commonly seen in female patients however in this study, we used male rats. We have not assessed the presence of LPS itself in the study since LPS is a short-lived molecule with higher intraindividual variability. Another limitation is that intestinal tissue investigation was not performed to show the intestinal barrier dysfunction. The results of this experimental rat study need to be further established in MOH patients.

## 5. Conclusion

Inflammation as shown by elevated brain IL-17 and HMGB1 levels and leaky gut as shown by increased serum occludin and LBP levels are possible mechanisms underlying MOH. It is very interesting and contrary to expectation, a nonsteroidal antiinflammatory agent to result in an inflammatory response. NSAIDs could increase intestinal permeability and cause leaky gut and result in chronic low-dose inflammation. MOH treatment is challenging, and it is hard to obtain sustained treatment outcomes in MOH patients. Taking into consideration the presence of leaky gut and LPS-induced inflammation in MOH could improve the treatment outcomes in those patients. Animal data may not be translated to humans all the time and studies in humans are required to confirm these findings. Treatments to reverse intestinal hyperpermeability should be considered in the management of MOH patients.
